# Frequency of Electrocardiographic Abnormalities in Tramadol Poisoned Patients; a Brief Report

**Published:** 2016

**Authors:** Anahita Alizadeh Ghamsari, Bita Dadpour, Fares Najari

**Affiliations:** 1 Faculty member of Mashhad University of Medical Sciences, Mashhad, Iran.; 2 Faculty member of the Shahid Beheshti University of Medical Sciences, Tehran, Iran.

**Keywords:** Tramadol, electrocardiography, arrhythmias, cardiac, drug-related side effects and adverse reactions, toxicity

## Abstract

**Introduction:**

Previous studies have raised the probably of cardiac manifestation in tramadol poisoning. However, conclusive information on electrocardiographic (ECG) abnormalities of tramadol overdose remains to be explained. Therefore, the present study aimed to evaluate the epidemiology of ECG abnormalities in tramadol poisoned patients.

**Methods:**

In a prospective cross-sectional study, all patients with tramadol poisoning, who were admitted to the emergency department of Loghman Hospital during 2012 – 2013, were evaluated. Patients’ baseline characteristics and ECG findings including axis, rate, rhythm, PR interval, QRS duration, QTc interval, evidence of Brugada pattern, and evidence of blocks were recorded. Obtained Data were descriptively analyzed using SPSS 21.0 statistical software.

**Results:**

1402 patients with the mean age of 24 ± 6 years were studied (71.1% male). Sinus tachycardia was detected in 463 (33%) patients, sinus bradycardia in one patient (0.07%), right axis deviation in 340 (24.2), QRS widening in 91 (6.5%), long QTc interval in 259 (18.4%), dominant S wave in either I or aVL lead in 395 (28.1%), and right bundle branch block in 73 (5.2%). Increased PR interval was not detected in any cases. The evidence of Brugada pattern was observed in 2 (0.14%) patients (100% male), both symptomatized with seizure. All abnormalities had same sex distribution.

**Conclusion:**

Based on the results of the present study, the most common types of ECG changes were sinus tachycardia, a deep S wave in leads I and aVL, right axis deviation, and long QTc interval, respectively. Brugada pattern and sinus bradycardia were rarely presented.

## Introduction

Tramadol is a synthetic centrally acting analgesic agent with opioid and non-opioid properties. Due to its relatively lower risk of respiratory depression or physical dependence and better safety profile in comparison with other opiates, it is extensively used for mild to moderate pain relief (1). The main adverse reactions of tramadol are nausea, dizziness, anorexia, and most importantly seizures and hypotension, which may occur in therapeutic or toxic doses (2). The drug is available, in both parenteral and oral pharmaceutical dosage forms; and has become one of the most widely dispensed analgesics in Iran’s essential drugs list since 2003. In recent years, tramadol abuse, misuse, and overdose have been dramatically increasing in Iran. Even international poisoning reports and suicide commitments by tramadol abuse have been increasing (-). Triad of opiate poisoning including miosis, respiratory depression, and decreased level of consciousness can be seen in tramadol overdose too, but unlike opioids, it leads to irritability, increased deep tendon reflexes, tremor, and hypertension. Other symptoms include urinary retention, rhabdomyolysis, seizures, and pulmonary edema (6, 7). As with many other drug toxicities, cardiac manifestations are also expected in tramadol poisoning. The cardiac symptoms may range from innocuous electrocardiographic (ECG) manifestations, such as sinus tachycardia, to life-threatening complications. It seems that tramadol overdose leads to some changes in the ECG through blocking of fast sodium and potassium channels (8). The study of ECG abnormality in 479 tramadol poisoned patients showed 30.6% sinus tachycardia, 24.6% QTC prolongation, and 31.7% QRS widening (9). There is a lack of information regarding possible effects of tramadol poisoning on the cardiovascular system and the most prevalent cardiac side effects of drug. Therefore, the present study was aimed to evaluate the epidemiology of ECG changes following tramadol poisoning in Iranian population. 

## Methods:

***Study Design and setting***

The present prospective cross-sectional study included tramadol poisoned patients who were admitted to emergency department (ED) of Loghman Hakim Hospital, Tehran, Iran, during one year from 2012 to 2013, using census sampling. The patients with co-ingestion, history of heart disease, history of dysrhythmias, as well as chronic tramadol users were excluded. A questionnaire consisting of demographic data (age, sex), vital signs (blood pressure, heart rate, respiratory rate, level of consciousness), time to ED arrival, dose of ingestion, history of previous use, drug history, comorbid diseases, cause of ingestion, and ECG findings (Axis, rate, rhythm, PR interval, QRS duration, QTc interval, evidence of Brugada pattern and blocks) were filled for all participants. ECGs were performed after physical examination by an emergency specialist. The protocol of the study was confirmed by Ethical Committee of Shahid Beheshti University of Medical Sciences. All authors adhered to Helsinki recommendations and confidentiality of patients’ data. All costs related to the project were incured by researchers, and no cost was imposed on patients. In addition, no changes were made in patients’ diagnosis and treatment process.

***Terms and definitions***

ECG abnormalities were defined as: sinus tachycardia (heart rate > 100 beats per minute), sinus bradycardia (heart rate < 60 beats per minute), increased PR interval (PR > 200 milliseconds), widening of the QRS duration (QRS ≥ 120 milliseconds), heart axis deviation (axis deviation in the frontal plane > 40 degree), R wave more than 1 millimeter in lead of aVL, and long corrected QT interval (QTc > 440 millimeter /second). Brugada pattern was defined as positive end of the QRS and ST-segment elevation in right precordial leads (V1 to V3). 

***Statistical analysis***

Data were descriptively analyzed using SPSS version 21 statistical software. Qualitative data were reported as frequency and percentage, and quantitative ones as mean ± standard deviation. 

## Results:

In this prospective cross-sectional study, 1402 patients with the mean age of 24 ± 6 years (14- 53) were studied (71.1% male). The baseline characteristics of patients are summarized in table 1. Sinus tachycardia was detected in 463 (33%) patients, sinus bradycardia in one patient (0.07%), right axis deviation in 340 (24.2), QRS widening in 91 (6.5%), long QTc interval in 259 (18.4%), dominant S wave in either I or aVL lead in 395 (28.1%), and right bundle branch block in 73 (5.2%) patients. Increased PR interval was not detected in any of the cases. The evidences of Brugada pattern was observed in 2 (0.14%) patients (100% male), both symptomatized with seizure. Table 2 shows the frequency of ECG abnormalities based on sex distribution.

**Table 1:** Baseline characteristics of the studied patients

**Variables**	**Number (%)**
**Age (years)**	
14 – 25	925 (66%)
26 – 35	393 (28 %)
36- 45	84 (6%)
**Sex**	
Male	997 (71.7%)
Female	405 (28.9%)
**Heart rate /minute**	
Mean ± SD	94 ± 12.94
**Blood pressure (mmHg)**	
< 90	191 (13.6%)
> 90	1211 (86.4%)
**Respiratory rate / minute**	
< 20	1052 (75%)
> 20	350 (25%)
**Level of Consciousness (GCS)**	
15	137 (9.8%)
< 15	1265 (90.2%)
**Time to ED arrival mean ± SD(hour)**	2 ± 0.5
**Dose of ingestion (mg)**	≥ 200
**Cause of ingestion**	
Intentional	400 (28.5%)
Un-intentional	1002 (71.5%)

GCS: Glasgow coma scale; SD: standard deviation.

**Table 2:** Frequency of ECG abnormalities based on sex distribution

**Abnormality**	**Number (%)**
**Sinus tachycardia (HR > 100 / minute)**	
Male	438 (94.6%)
Female	25 (5.4%)
**QRS interval widening (≥ 120 millisecond)**	
Male	64 (70.3%)
Female	27 (29.7%)
**Long QTc interval (≥ 440 millisecond)**	
Male	134 (51.7%)
Female	125 (48.3%)
**Right axis deviation ( > 40 degree)**	
Male	259 (76.2%)
Female	81 (23.8%)
**Dominant S wave ** **(in I or aVL leads)**	
Male	372 (94.2%)
Female	23 (5.8%)
**Right bundle branch block**	
Male	40 (54.8%)
Female	33 (45.2%)

## Discussion:

Based on the results of the present study, the most common types of ECG changes were sinus tachycardia (33%), a deep S wave in leads I and aVL (28%), right axis deviation (24%) and long QTc interval (18%), respectively. Brugada pattern and sinus bradycardia were rarely presented. 

Most studies concerning complications of tramadol overdose, have described central nervous system (CNS) manifestations of the drug and seizure (-). There have been only few reports on cardiovascular toxicities including ECG abnormalities (-). Emamhadi et al. first described ECG manifestations of tramadol overdose in a retrospective study. Our results are very similar to those obtained by Emamhadi’s work on 479 patients poisoned by tramadol. Their findings demonstrated occurrence of tachycardia in 30.6%, long QRS duration in 7.5%, QTc > 440 millimeter/second in 24.6%, R wave height more than 1 mm in aVR lead in 22.1%, right axis deviation in 31.7%, and complete or incomplete right bundle branch block in 4.6% of patients. They concluded that tramadol overdose, through fast blockade of sodium and potassium channels, leads to some changes in the ECG, which potentially may progress to life-threatening arrhythmias (9)

Sinus tachycardia was observed in a significant number of patients (33%) the majority of which were male (94%). Although sinus tachycardia is usually non-specific for opioids, and bradycardia is a more common finding with other opioids (11, 18), our findings are in line with some other studies focusing on tramadol. In a mult-icenter prospective case series study of patients with tramadol overdose, Spiller et al. observed tachycardia in 13% of patients (12). Marquardt and colleagues also reported 17.4% tachycardia (19). However, in comparison with previously performed studies, the number of patients in the current study was vastly higher, although the sex ratio was not very different from other studies. This is largely because the incidence of drug poisoning in Iran is not comparable to Western countries. Tramadol in Iran is more easily and readily available compared to Western countries (3). 

Brugada pattern was seen in only 2 patients, both were male. In the study by Emamhadi et al., there was one patient (among 479) with Brugada pattern (9). There was also one isolated case report of Brugada pattern by Cole JB, which believed that sodium-channel blockade effect of tramadol was responsible for this complication of the drug (15). Tramadol has been previously shown to reversibly block sodium channels especially at high concentrations. However, it is unclear that Brugada syndrome existed before tramadol poisoning or is one of its complications (15, 20). 

 QTc prolongation was also a common ECG abnormality among our patients that may also be attributed to rapid blockade of sodium channels. Yet, it may also be due to a probable potassium channel blockade since tramadol has been previously shown to have inhibitory effects on potassium channels (-). QTc prolongation is the primary ECG manifestation of potassium channel blockers (8). QRS widening, seen in few patients, was also reported by Emamhadi et al. in 7% of their patients.

In this study, like other studies, no relationship between the ECG changes and amount of drug used or history of drug use was observed. 

Finding the accurate pattern of ECG abnormality(s) of tramadol poisoning needs further multi-center studies with larger sample size. In addition, study on pharmacologic characteristics of tramadol could be helpful in discovering its mechanism of cardiac effect and ECG abnormalities that follow.

## Conclusion:

Based on the results of the present study, the most common types of ECG changes were sinus tachycardia, a deep S wave in leads I and aVL, right axis deviation, and long QTc interval, respectively. Brugada pattern and sinus bradycardia were rarely presented. 

## References

[1] Vazzana M, Andreani T, Fangueiro J, Faggio C, Silva C, Santini A (2015). Tramadol hydrochloride: pharmacokinetics, pharmacodynamics, adverse side effects, co-administration of drugs and new drug delivery systems. Biomedicine & pharmacotherapy = Biomedecine & pharmacotherapie.

[2] Beakley BD, Kaye AM, Kaye AD (2015). Tramadol, Pharmacology, Side Effects, and Serotonin Syndrome: A Review. Pain physician.

[3] Iravani FS, Akhgari M, Jokar F, Bahmanabadi L (2010). Current trends in tramadol-related fatalities, Tehran, Iran 2005–2008. Substance use & misuse.

[4] Hassanian Moghaddam H, Noroozi A, Zafaghandi S, Bagher M, Sarjami S (2015). Substance abuse warning network: Pilot results in poisoned patients. Razi Journal of Medical Sciences.

[5] Organization WH, Dependence WECoD (2015). WHO Expert Committee on Drug Dependence: thirty-sixth report.

[6] Mehrpour O, Sharifi M, Zamani N (2015). Tramadol Poisoning.

[7] Shadnia S, Soltaninejad K, Heydari K, Sasanian G, Abdollahi M (2008). Tramadol intoxication: a review of 114 cases. Human & experimental toxicology.

[8] Lionte C, Bologa C, Sorodoc L (2012). Toxic and drug-induced changes of the electrocardiogram.

[9] Emamhadi M, Sanaei-Zadeh H, Nikniya M, Zamani N, Dart RC (2012). Electrocardiographic manifestations of tramadol toxicity with special reference to their ability for prediction of seizures. The American journal of emergency medicine.

[10] Asadi P, Monsef Kasmaei V, Ziabari SZ, Zohrevandi B, Moadab Manesh A (2015). Prevalence of Tramadol Consumption in First Seizure Patients; a One-Year Cross-sectional Study. Emergency.

[11] Afshari R, Afshar R, Mégarbane B (2011). Tramadol overdose: review of the literature. Réanimation.

[12] Spiller HA, Gorman SE, Villalobos D, Benson BE, Ruskosky DR, Stancavage MM (1997). Prospective multicenter evaluation of tramadol exposure. Journal of toxicology Clinical toxicology.

[13] Tobias JD (1997). Seizure after overdose of tramadol. Southern medical journal.

[14] Afshari R, Ghooshkhanehee H (2009). Tramadol overdose induced seizure, dramatic rise of CPK and acute renal failure. JPMA The Journal of the Pakistan Medical Association.

[15] Cole JB, Sattiraju S, Bilden EF, Asinger RW, Bertog SC (2012). Isolated tramadol overdose associated with Brugada ECG pattern. Pacing and clinical electrophysiology : PACE.

[16] Garrett PM (2004). Tramadol overdose and serotonin syndrome manifesting as acute right heart dysfunction. Anaesthesia and intensive care.

[17] Daubin C, Quentin C, Goulle JP, Guillotin D, Lehoux P, Lepage O (2007). Refractory shock and asystole related to tramadol overdose. Clinical toxicology.

[18] Chen A, Ashburn MA (2015). Cardiac Effects of Opioid Therapy. Pain medicine (Malden, Mass).

[19] Marquardt KA, Alsop JA, Albertson TE (2005). Tramadol exposures reported to statewide poison control system. The Annals of pharmacotherapy.

[20] Haeseler G, Foadi N, Ahrens J, Dengler R, Hecker H, Leuwer M (2006). Tramadol, fentanyl and sufentanil but not morphine block voltage-operated sodium channels. Pain.

[21] Yalcin I, Aksu F (2005). Involvement of potassium channels and nitric oxide in tramadol antinociception. Pharmacology, biochemistry, and behavior.

[22] Cho HC, Sohn JT, Park KE, Shin IW, Chang KC, Lee JW (2007). Inhibitory effect of tramadol on vasorelaxation mediated by ATP-sensitive K+ channels in rat aorta. Canadian journal of anaesthesia = Journal canadien d'anesthesie.

[23] Tsai TY, Tsai YC, Wu SN, Liu YC (2006). Tramadol-induced blockade of delayed rectifier potassium current in NG108-15 neuronal cells. European journal of pain.

